# New species of *Plectromacronema* Ulmer 1906 (Trichoptera: Hydropsychidae) from Brazil, with association of immature stages and aspects of its biology

**DOI:** 10.1371/journal.pone.0197573

**Published:** 2018-06-13

**Authors:** Henrique Paprocki, Larissa Moreira-Silva

**Affiliations:** Pontifícia Universidade Católica de Minas Gerais, Belo Horizonte, Minas Gerais, Brazil; National Cheng Kung University, TAIWAN

## Abstract

*Plectromacronema* Ulmer 1906 is a Neotropical genus recorded sparsely from South of Mexico to the North of Argentina. The genus is placed within the Macronematinae sub-family, members of which are relatively large among the Trichoptera and bears conspicuous wing ornamentations. There are only two species recorded for Brazil, *Plectromacronema comptum* Ulmer 1906 and *Plectromacronema subfuscum* (Banks 1920). *Plectromacronema lisae* Flint 1983 recorded for Mexico and Costa Rica is the only species in the genus with immature stages described. Immature stages species level identification is required for water quality biomonitoring and most of the Trichoptera taxonomy is based only on adult male genitalia. In this paper, we propose a new species, *Plectromacronema solaris* sp.nov., larva, pupa, and adult. Aspects of the life history of the new species are also discussed. This record also represents a new genus record for the state of Minas Gerais, extending the knowledge on the diversity, distribution, biogeography, and biology of this remarkable genus.

## Introduction

Trichoptera is the largest order of exclusively aquatic insects, having nearly 14,500 species described currently [1]. There are 3,262 species recorded for the Neotropical region [2] and 625 recorded for Brazil [3]. The family Hydropsychidae Curtis, 1835 belongs to the suborder Annulipalpia which is also known as the net spinning Trichoptera. Hydropsychidae is the third largest Trichoptera family with 1,820 described species placed in 39 genera worldwide [4]. Hydropsychidae is the most diverse among the families present in Brazil with 124 species recorded for the country [3]. The family is divided into five subfamilies: Arctopsychinae Martynov 1924, Diplectroninae Ulmer 1951, Hydropsychinae Curtis 1835, Macronematinae Ulmer 1905a, and Smicrideinae Flint 1974 [2,5], all present in the Neotropics except for Arctopsychinae. Only two subfamilies are found in Brazil: Macronematinae with the genera *Blepharopus*, *Centromacronema*, *Leptonema*, *Macronema*, *Macrostemum*, *Plectromacronema*, and *Synoestropsis* genera and Smicrideinae with only the genus *Smicridea* [6].

**Fig 1:**
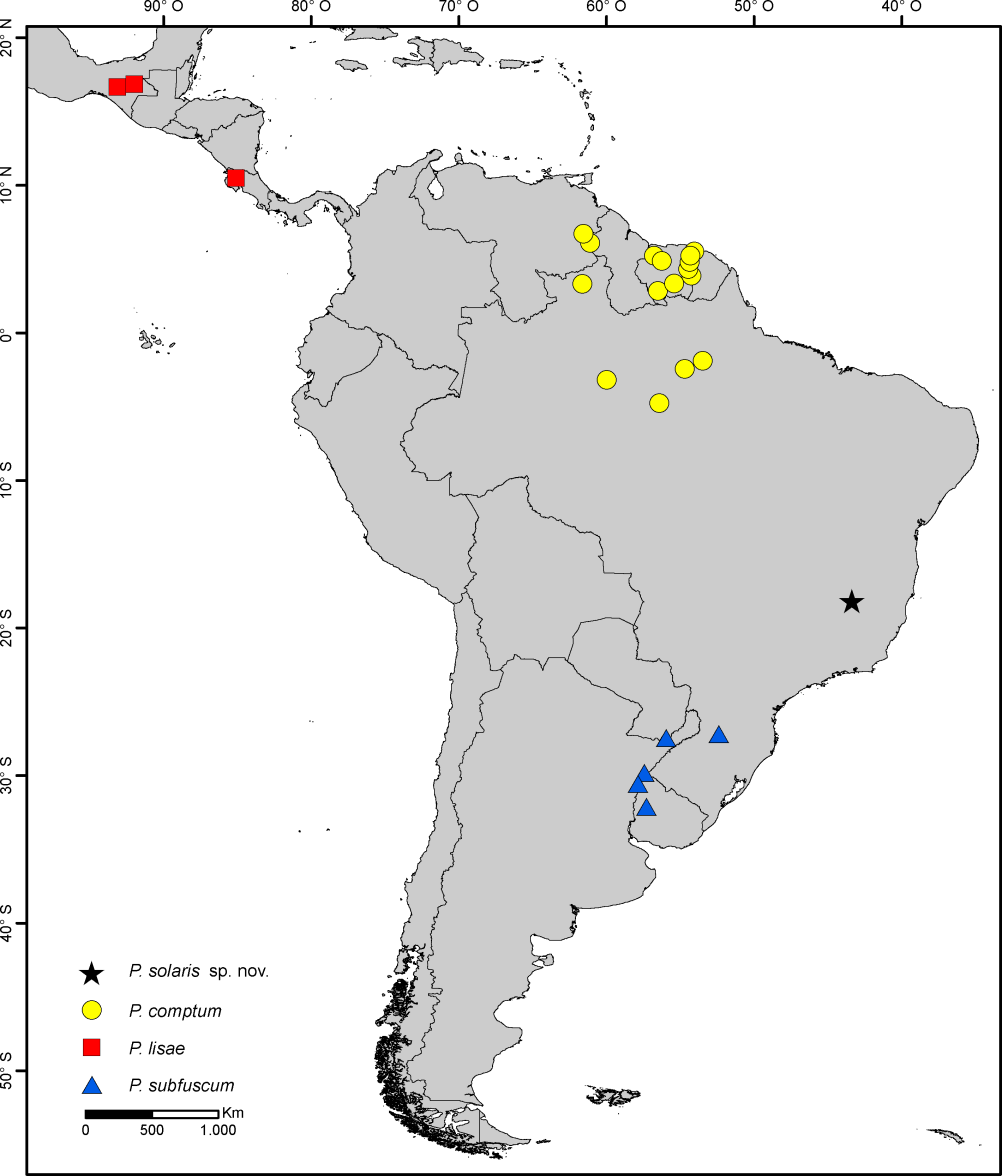
Distribution of *Plectromacronema* species and type locality of *Plectromacronema solaris* sp.nov. Rio Preto State Park, Minas Gerais, Brazil.

*Plectromacronema* is a Neotropical genus revised by Flint in 1983 [7], holding 3 described species: *P*. *comptum* Ulmer 1906 [8], *P*. *lisae* Flint 1983 [7] and *P*. *subfuscum* (Banks) 1920 [9]. The species *P*. *comptum* is recorded for the Brazilian states of Amazonas [8,10,11], Roraima [12], and Pará [11] and for the countries of Surinam [13], French Guiana [14], Venezuela [7,15], and Guyana [16]. The species *P*. *subfuscum* is recorded from the Brazilian state of Santa Catarina [7] and the countries Argentina [9,17] and Uruguay [7]. The species *P*. *lisae* is recorded from Costa Rica and Mexico with immature stages and habits described by Flint (1983) [7] (Fig 1).

The species level identification of the immature stages of aquatic insect is crucial for ecological and evolutionary studies because most of their life cycle happens under water [2]. The progress of water quality biomonitoring and ecological studies has been impeded in the Neotropics because most of the species immature stages are unknown [18].

## Material and methods

**Fig 2:**
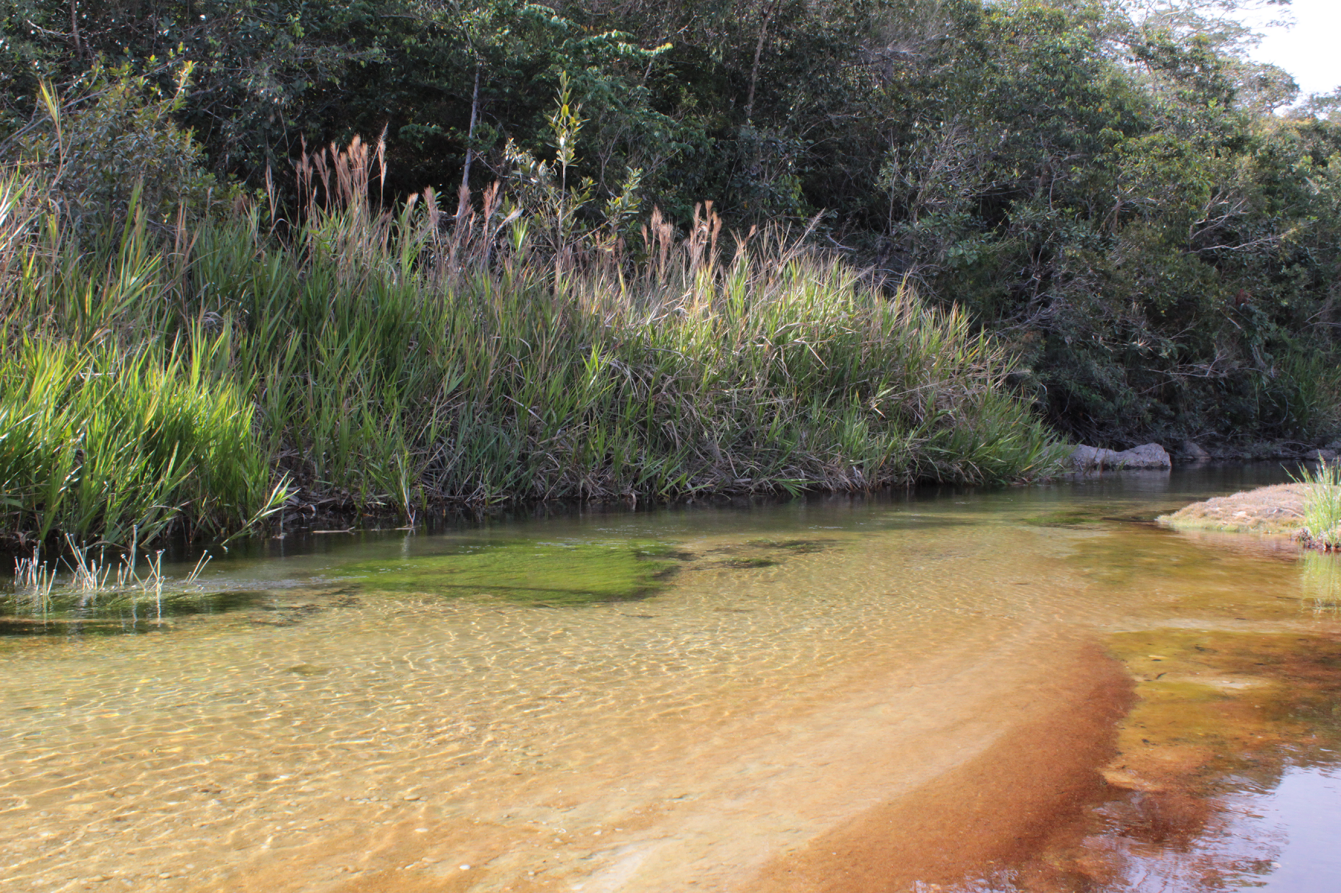
*Plectromacronema solaris* sp. nov. Habitat, Rio Preto stream stretch where part of type series was collected.

The Trichoptera population studied is found in Parque Estadual do Rio Preto, a state conservation area and the type locality of the new species (Fig 1). The Park (18°7'7"S 43°20'41"W) is located in the municipality of São Gonçalo do Rio Preto state of Minas Gerais and it has a total area of 12,184 hectares. The park is mostly of cerrado (Brazilian seasonal savanna), with rock fields and riparian forests along the watercourses. The streams’ bottom is mostly of boulders and large gravel in the rapids and sand in the pools. Large pools with more than 50m of diameter are commonly found in the sampled stretch. The rapids are usually up to 5m wide when the bank is full (Fig 2). The waters are dark, stained by humic acids, a common characteristic of the Espinhaço mountain range [19], where the park is located. Flash floods in the rainy season and reduced, but perennial, flow in the dry season are the rule of thumb for the local streams.

The larvae were mostly found in slow current stretches. They can be seen from outside the water because of the tube shaped retreat that emerges 2–4 cm from the substrate. The larvae were collected manually taking each one individually from the sandy substrate using a sieve or holding both hands as a scoop. Larvae were preserved in 80% ethanol or brought alive to the laboratory where they were reared. Larvae brought to the laboratory on 24/10/2015 were reared in a 32-liter water tank with sand in the bottom and a pump keeping a slow current recirculating the water. The substrate and the water used in the tank were brought from the collection site. The water tank was refilled with generic spring bottled water. Larvae were fed individually with *Nauphoeta cinerea* (Olivier) 1798, flour (dehydrated and finely grated roaches) every other day with a small portion held in a fine tip forceps at the opening of the retreat. The immature/adult stages association was acquired through direct rearing in the laboratory.

The adults were attracted to an ultraviolet light hanging in front of a white sheet set by the shore of the streams at dusk and killed with a potassium cyanide kill jar. Association of males and females were established through specimens emerged from the water tank in the laboratory. The adults were pinned in entomological pins and labeled for localities.

The abdomen of the adult specimens were sectioned between segment 6–8, placed in glass tubes containing 85% lactic acid; and then heated up to 110°C for approximately 50 minutes. The already cleansed genitalia were transferred to a container containing distilled water to rinse and later stored in alcohol 80% [20].

Detailed examination of the larvae and genitalia dissected and cleared were performed, followed by pencil sketches done using a camera lucida mounted on an Olympus SZX12 stereoscopic microscope. The illustrations were then rendered using Adobe Illustrator CS6. A scanning electron microscope JEOL JSM-IT 300 was used for examination of structures and retreats. The morphological nomenclature was adapted from Flint 1983 [7], Flint et al. [21], and Holzenthal 2007 [22]. The wings color patterns were compared with photographs taken from *P*. *comptum* holotype and literature Ulmer 1906, Ulmer 1913, Flint 1983 [7,8,17].

Part of the examined material is deposited in the entomological collection, Museu de Ciências Naturais da Pontifícia Universidade Católica de Minas Gerais (MCN-PUC Minas). The type series is deposited at Museu de Zoologia da Universidade de São Paulo (MZUSP).

### Nomenclatural acts

The electronic edition of this article conforms to the requirements of the amended International Code of Zoological Nomenclature, and hence the new names contained herein are available under that Code from the electronic edition of this article. This published work and the nomenclatural acts it contains have been registered in ZooBank, the online registration system for the ICZN. The ZooBank LSIDs (Life Science Identifiers) can be resolved and the associated information viewed through any standard web browser by appending the LSID to the prefix “http://zoobank.org/”. The LSID for this publication is: urn:lsid:zoobank.org:pub:24497F1D-286F-4902-A685-F82542940B73. The electronic edition of this work was published in a journal with an ISSN, and has been archived and is available from the following digital repositories: PubMed Central, LOCKSS.

## Taxonomy

The genus *Plectromacronema* was described by Ulmer in 1906 [8] with the type species *Plectromacronema comptum*. Banks in 1920 [9] described a new monotypic genus for *Podomacronema subfuscum*. Following, Flint in 1967 [23] examined the holotypes of both species and decided to apply a generic synonym to *Podomacronema* with *Plectromacronema*. *Plectromacronema lisae* was described by Flint in 1983 [7] and in this paper, more than 3 decades later, a new species of this remarkable Neotropical genus is presented. The diagnostic characteristics of the genus proposed by Ulmer [8], Banks [9] and Flint [23] are selected and summarized as follows: adult head with an elevated dome shaped vertex; apex of front wing with a conspicuous folding near the M1 vein. Larvae with segments 2–8 with paired ventrolateral pockets, densely covered internally with hooked spines; lateral line present on abdominal segments 4–8; larvae inhabiting tubular retreats built out of silk and sand grains without a capture net.

The genus evidently demonstrates a vicariant distribution (Fig 1). *Plectromacronema lisae* is restricted to Central America watersheds, while *P*. *comptum* is found in central Amazonia, and Guianas in the northern portion of South America, yet *P*. *subfuscum* is distributed in La Plata river tributaries, subtropical South America. *Plectromacronema solaris* sp. nov. is found in tributaries of Rio Jequitinhonha, in one of the Brazilian eastern Atlantic watersheds, more than one thousand kilometers from the closest known species records of *Plectromacronema*.

### Species catalog

#### *Plectromacronema comptum* Ulmer 1906

Ulmer, 1906:63 [Description][8]; Ulmer, 1907:41 [Wing venation, head illustration][10]; Ulmer, 1913:392 [Misidentification- Ulmer describes *P*. *subfuscum* as *P*. *comptum*][17]; Fischer, 1963:163 [Catalog][24]; Flint, 1974:115 [Genitalia][13]; Flint, 1978:395–403 [Distribution][11]; Flint, 1983:226 [Distribution, wing color pattern, genitalia][7]; Flint,1991:69 [Distribution][12]; Flint, Holzenthal and Harris, 1999:70 [Catalog][6]; Paprocki, Holzenthal and Blahnik, 2004:9 [Checklist][25]; J.Olah and K A Johanson, 2012:242 [Distribution][14]; Paprocki and França, 2014:31 [Checklist][3]; Holzenthal and Calor, 2017:158 [Catalog][2].

**Holotype**: Brazil, Pará, Santarém, British Museum of Natural History (BMNH), Male.

**Distribution**: Brazil, French Guiana, Guyana, Suriname, Venezuela (Fig 1).

#### *Plectromacronema lisae* Flint 1983

Flint, 1983:228 [Description larva, pupa, adult][7]; Holzenthal, 1988:69 [Distribution][26]; Flint, Holzenthal and Harris, 1999:70 [Catalog][6]; Bueno-Soria and Barba-Álvarez, 2011:356 [Checklist][27]; Holzenthal and Calor, 2017:158 [Catalog][2].

**Holotype**: Mexico, Chiapas, National Museum of Natural History (NMNH), Male.

**Distribution**: Costa Rica, Mexico (Fig 1).

#### *Plectromacronema subfuscum* (Banks 1920)

Banks, 1920:356 [Description in *Podomacronema*][9]; Ulmer, 1913:392 [Misidentification- Distribution, wing venation, wing color pattern][17]; Fischer, 1963:164 [Catalog in *Podomacronema*][24]; Flint,1967:11 [Synonymized to *Plectromacronema*][23]; Flint, 1983:227 [Wing color pattern, genitalia, distribution][7]; Flint, Holzenthal and Harris, 1999:70 [Catalog][6]; Paprocki, Holzenthal and Blahnik, 2004:9 [Checklist][25]; Paprocki and França, 2014:31 [Checklist][3]; Holzenthal and Calor, 2017:158 [Catalog][2].

**Holotype**: Argentina, Misiones, Museum of Comparative Zoology (MCZ), Male.

**Distribution**: Argentina, Brazil, Uruguay (Fig 1).

### New species

#### *Plectromacronema solaris* sp. nov.

urn:lsid:zoobank.org:act:1BF64DF5-5665-4E6B-8900-08A9EC145D78

(Figs 3–12)

**Fig 3:**
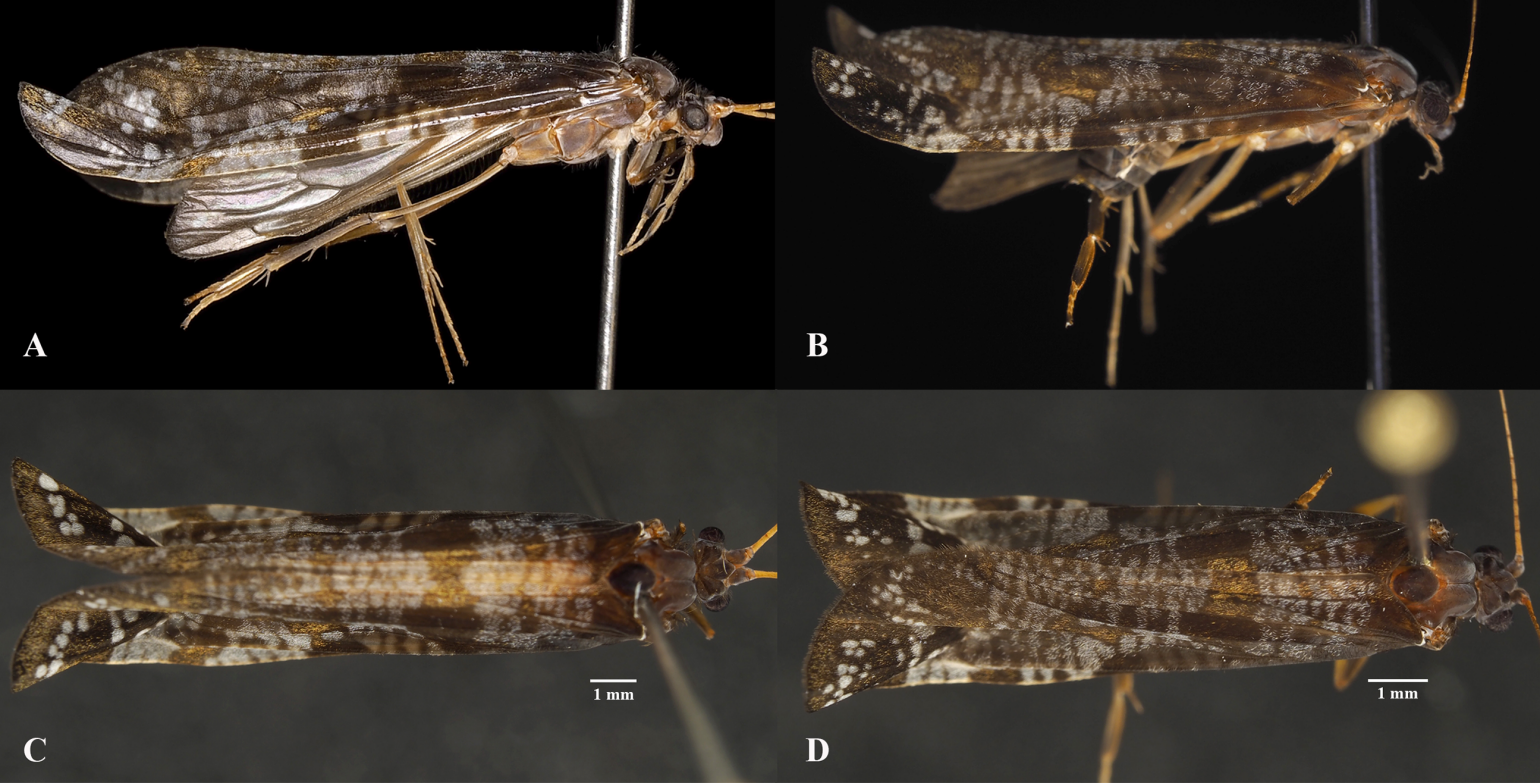
*Plectromacronema solaris* sp. nov. Full habitus. A, male lateral. B, female lateral. C, male dorsal. D, female dorsal.

**Fig 4:**
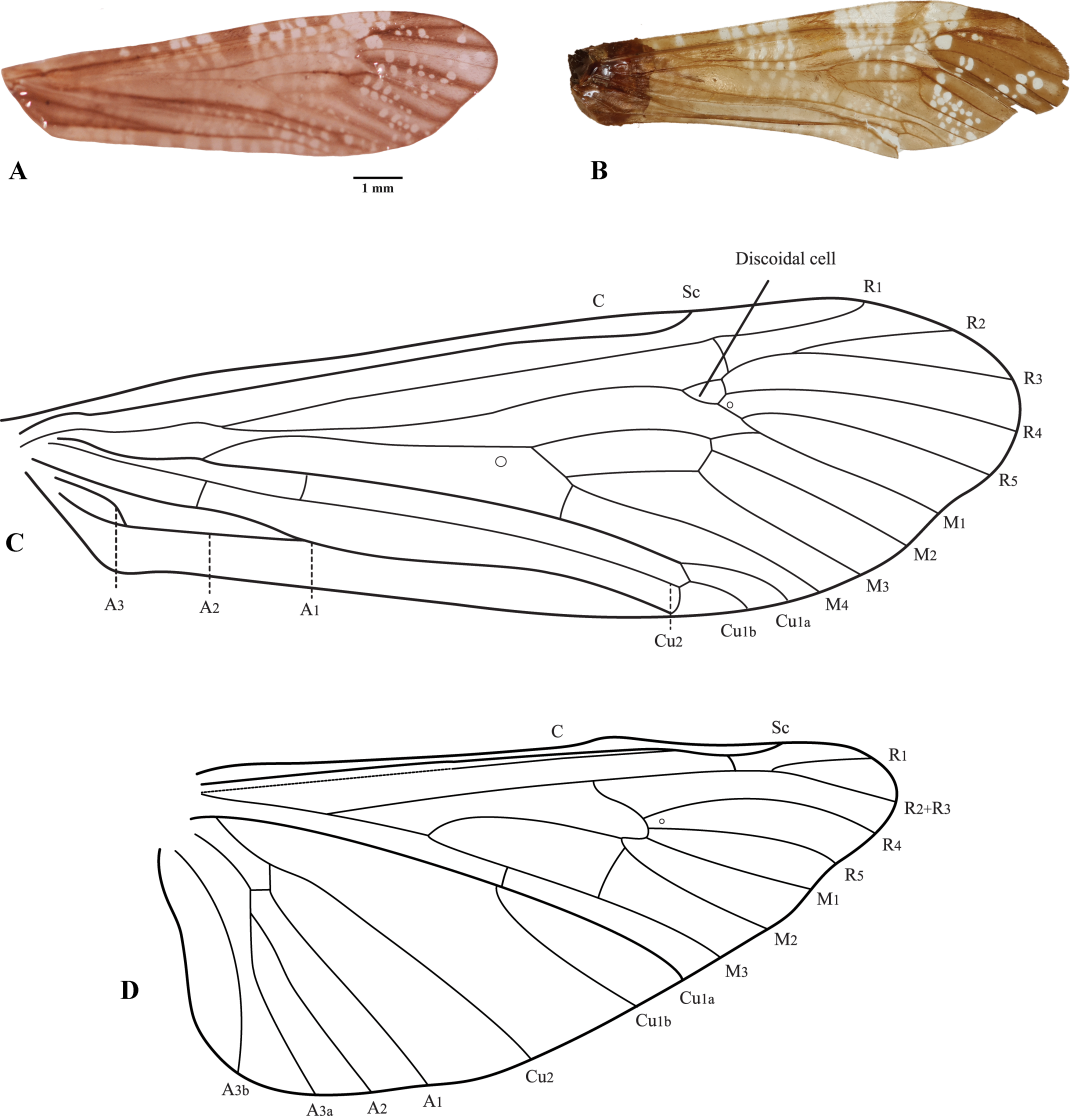
*Plectromacronema solaris* sp. nov. Wings. A, forewing color markings of *Plectromacronema solaris* sp. nov. B, forewing color markings from holotype of *Plectromacronema comptum*. C, forewing venation of *Plectromacronema solaris* sp. nov. D, hindwing venation of *Plectromacronema solaris* sp. nov. Abbreviation of the wing veins: C, costa. Sc, subcosta. R, radius. M, media. Cu, cubitus. A, anal.

**Fig 5:**
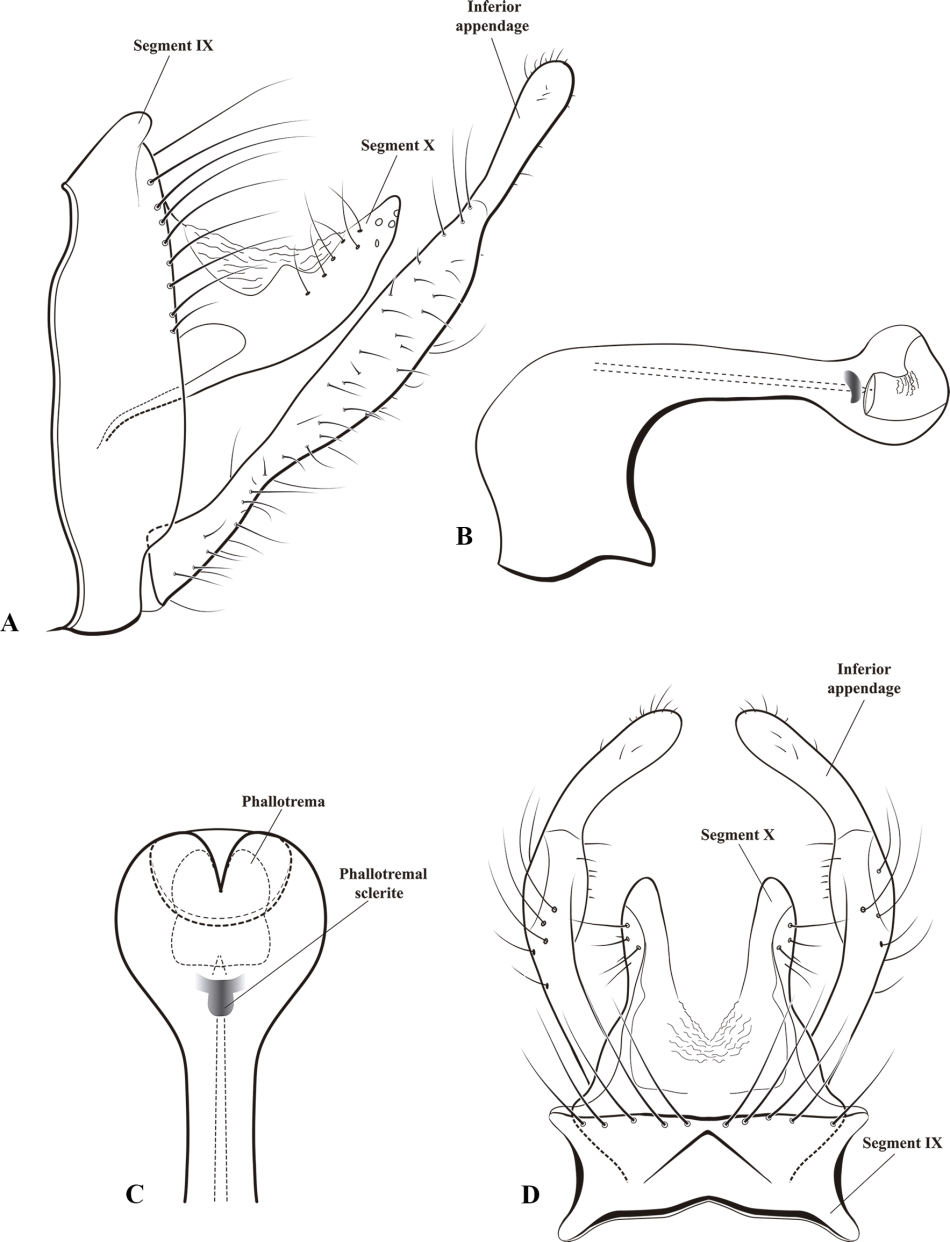
*Plectromacronema solaris* sp. nov. Male genitalia. A, lateral view. B, phallus lateral. C, phallus apex ventral. D, dorsal view.

**Fig 6:**
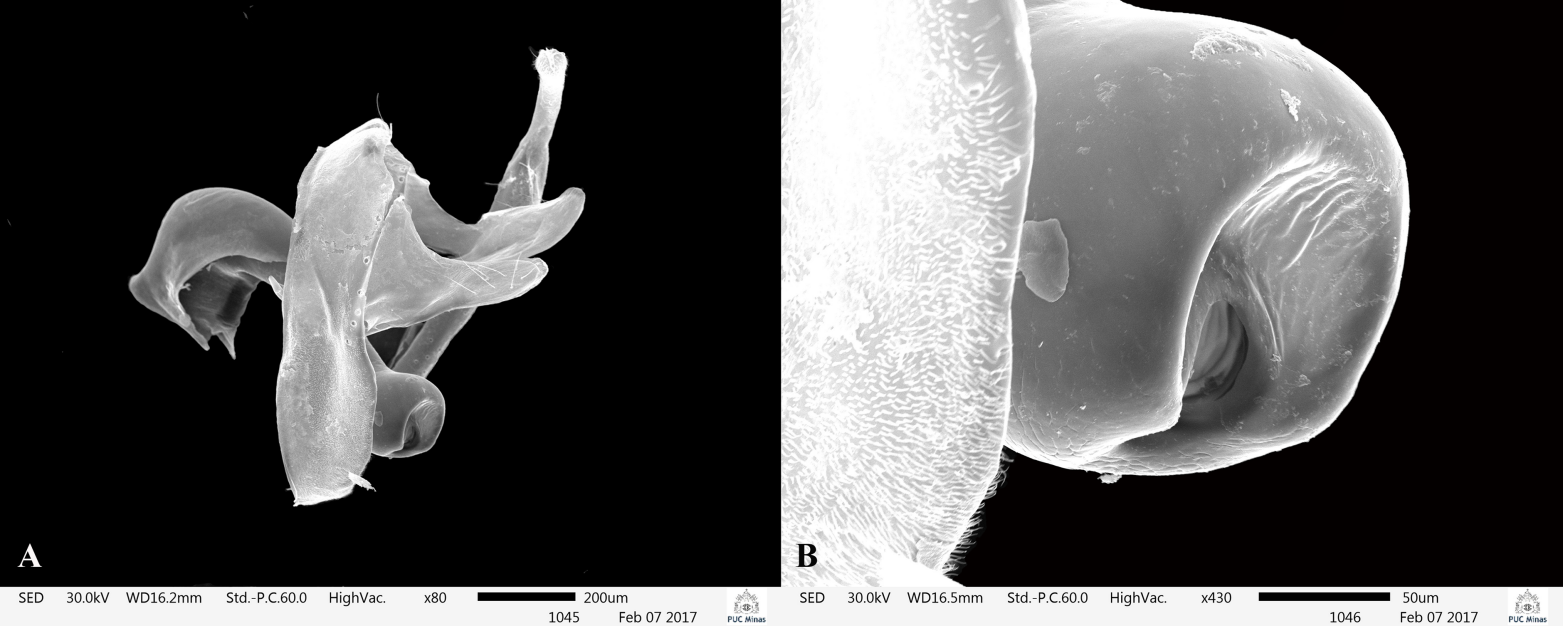
*Plectromacronema solaris* sp. nov. Scanning Electron Microscopy, male genitalia. A, IXth and Xth abdominal segments and phallus. B, phallus apex.

**Fig 7:**
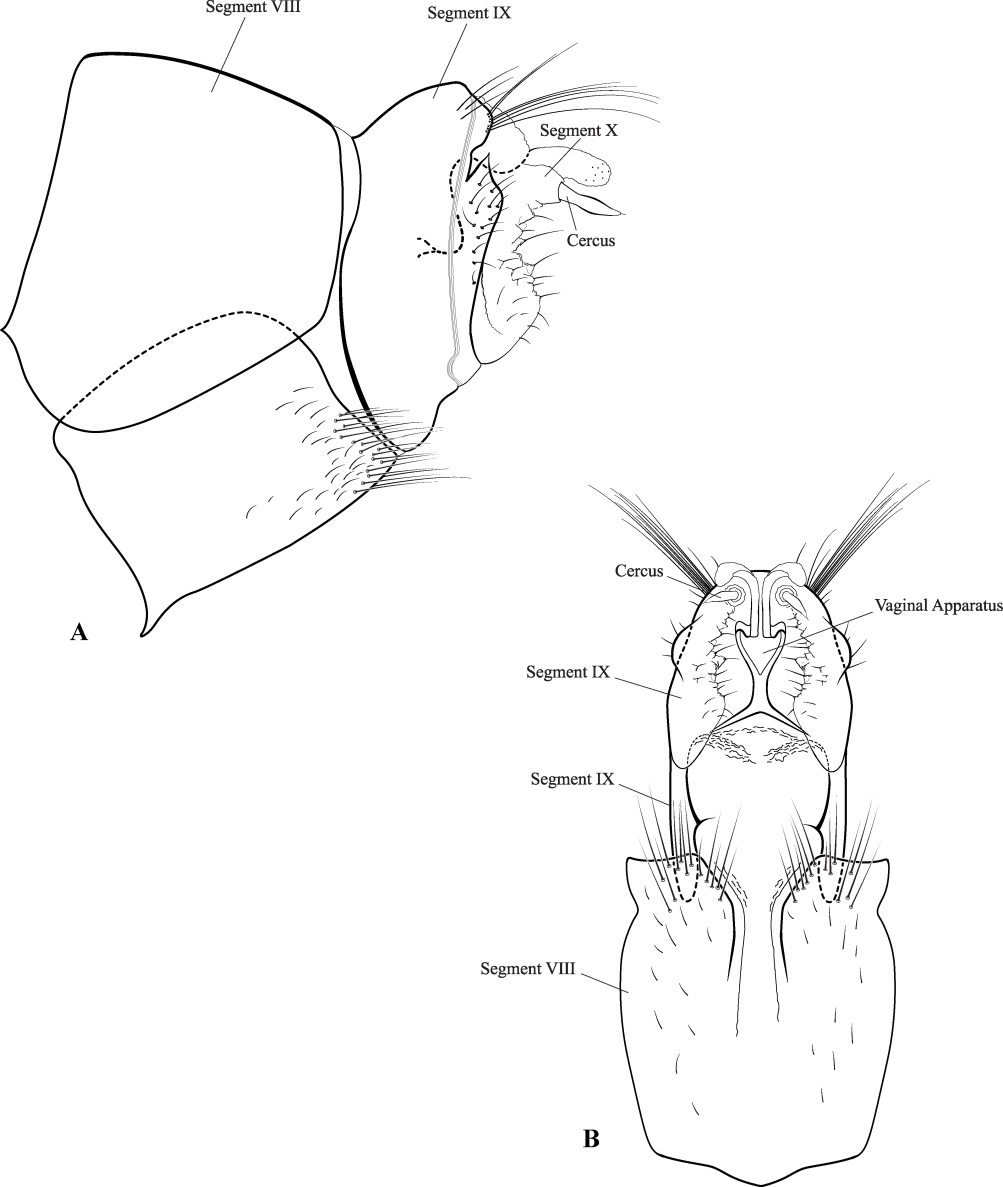
*Plectromacronema solaris* sp. nov. Female genitalia. A, lateral view. B, ventral view.

**Fig 8:**
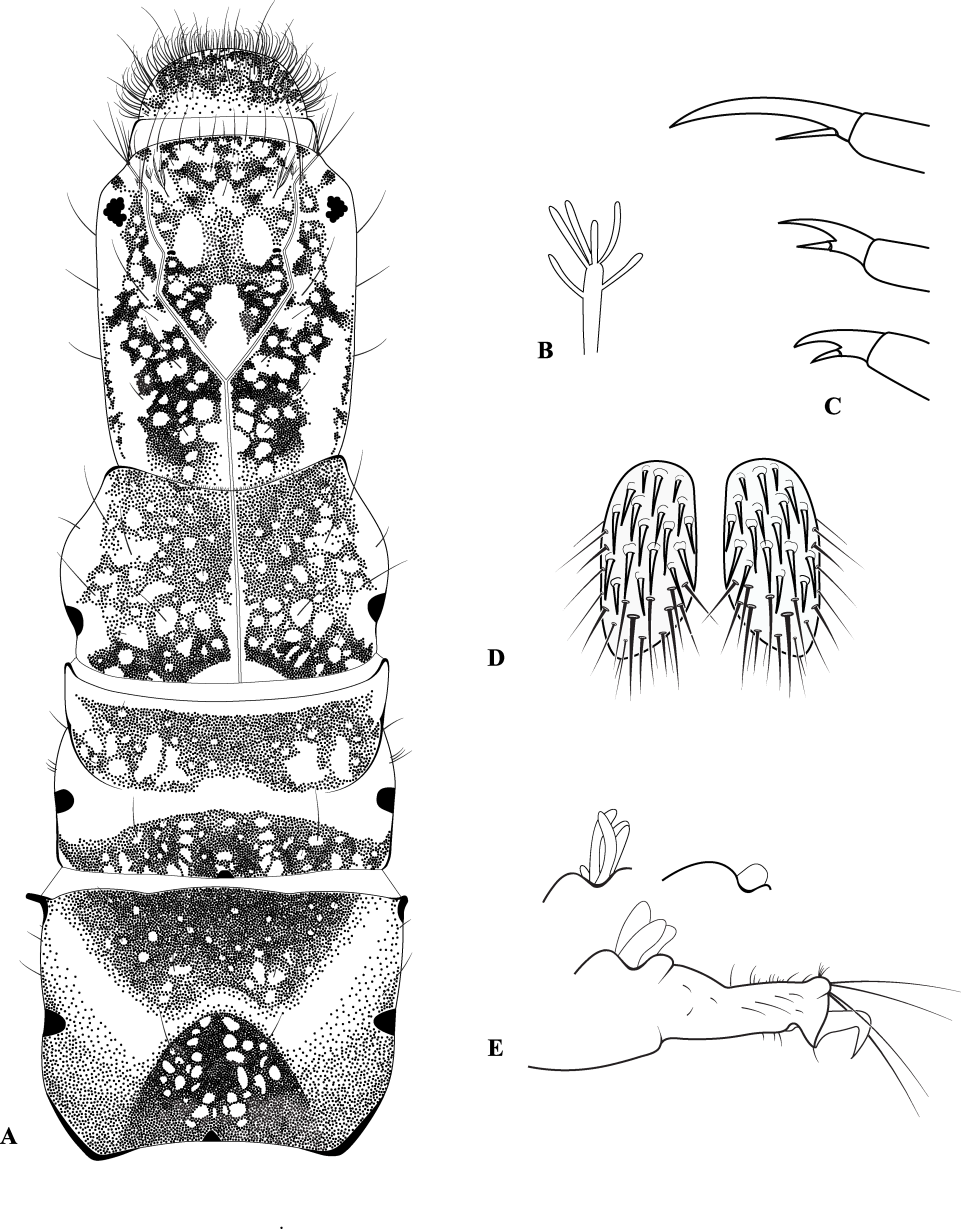
*Plectromacronema solaris* sp. nov. Larva. A, dorsal view, head and thorax. B, ventral gills. C, tarsal claws. D, ventral sclerite in abdominal sternum IX. E, anal proleg lateral view with anal papillae variations in detail above.

**Fig 9:**
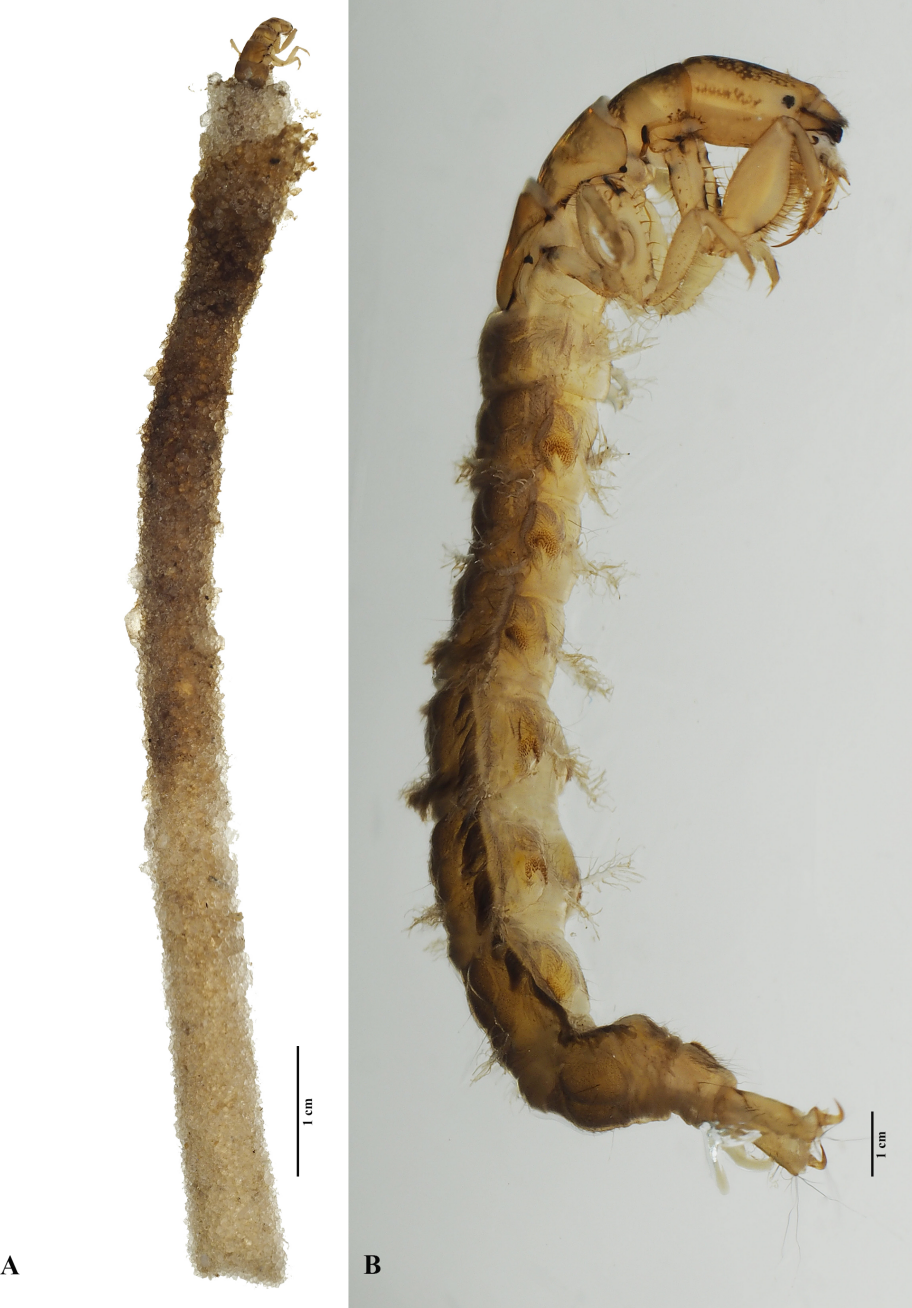
*Plectromacronema solaris* sp. nov. Larva. A, case with larva, lateral view. B, larva lateral view.

**Fig 10:**
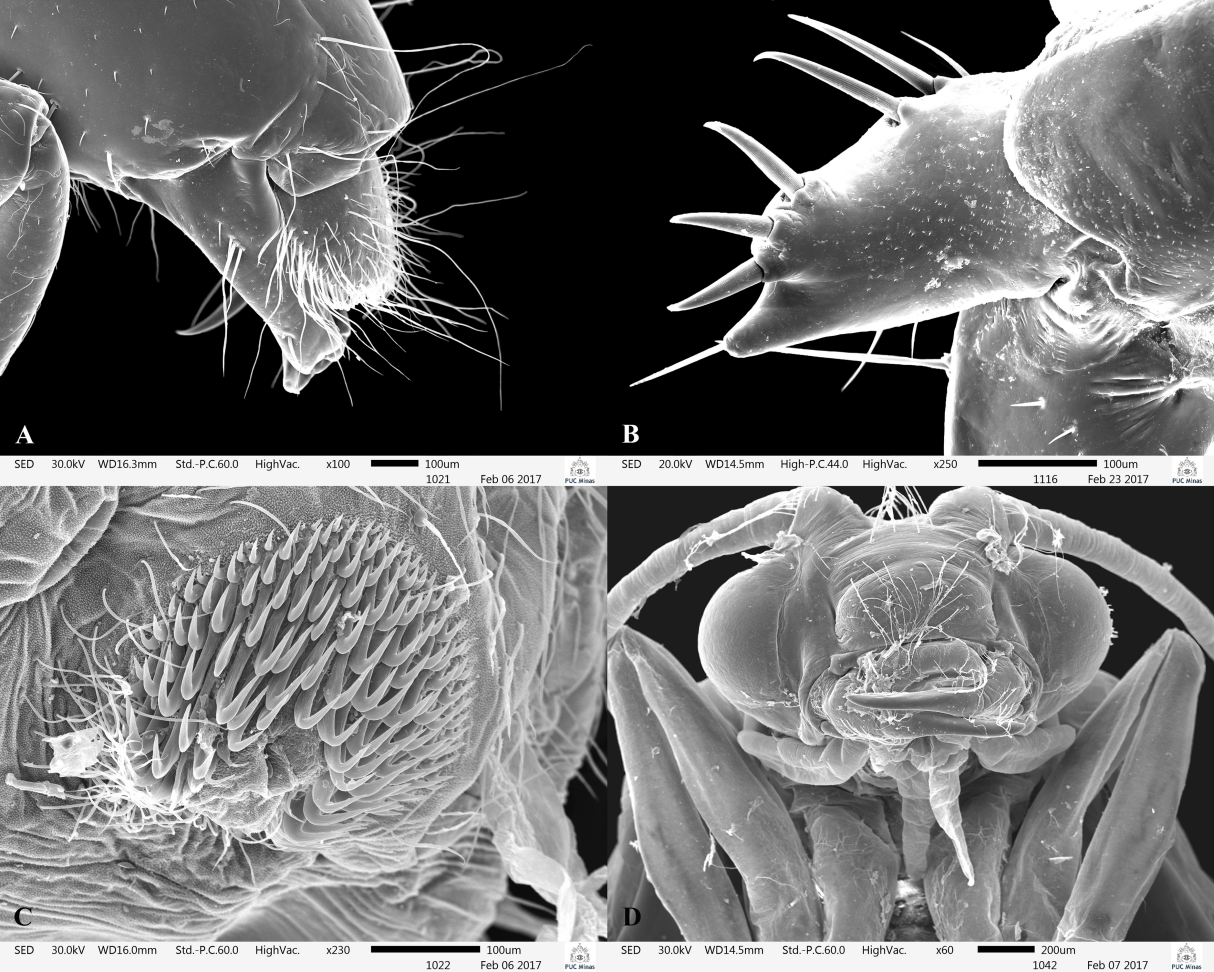
*Plectromacronema solaris* sp. nov. Scanning Electron Microscopy larva and pupa. A, larva mouthparts lateral. B, larva foretrochantin, lateral. C, larva abdominal hook pocket. D, pupal head, ventral view.

**Fig 11:**
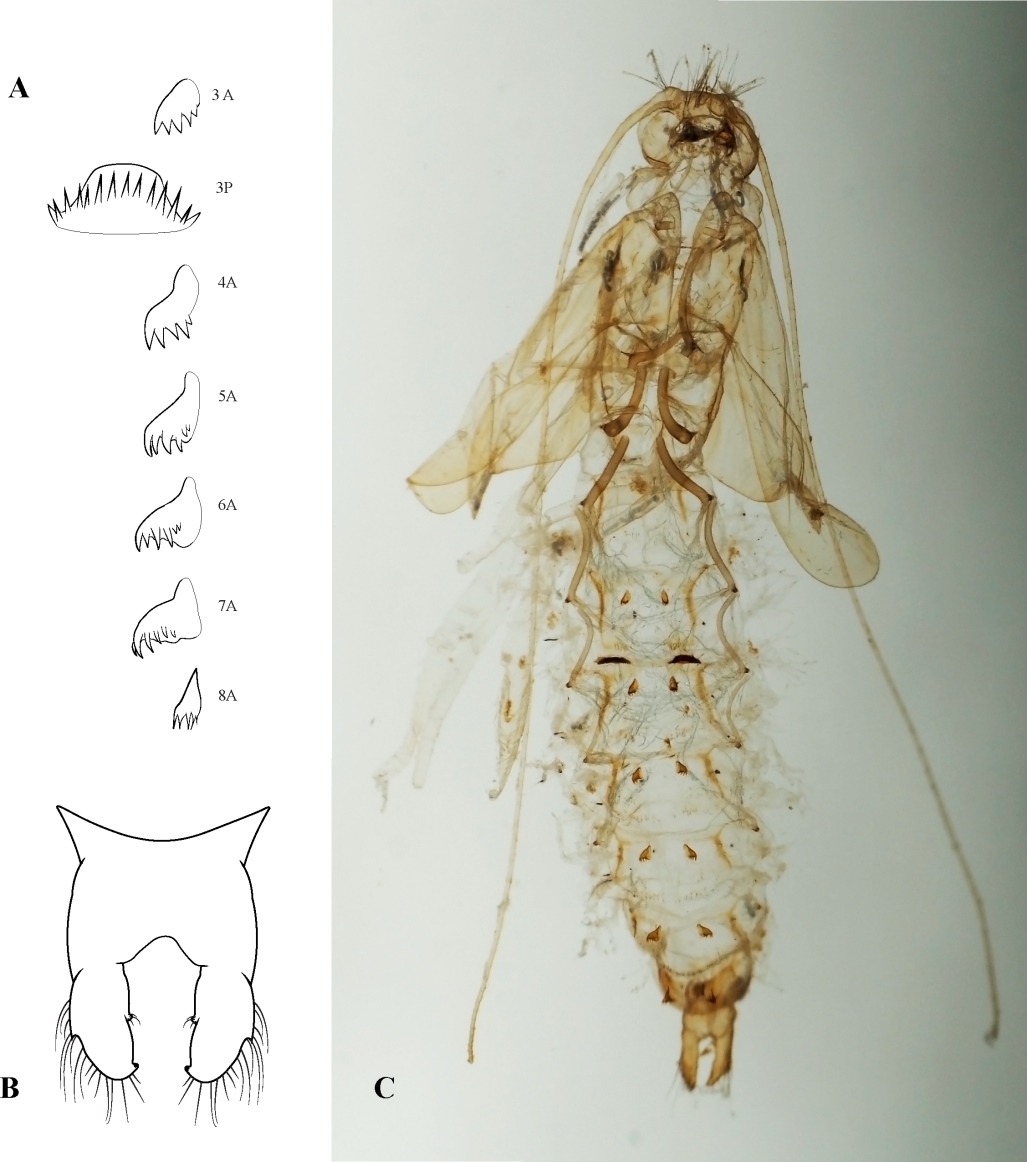
*Plectromacronema solaris* sp. nov. Pupa. A, hook plates, numbers followed by letter represent abdominal segments and anterior or posterior position. B, pupal apical appendage. C, pupal exuviae.

**Fig 12:**
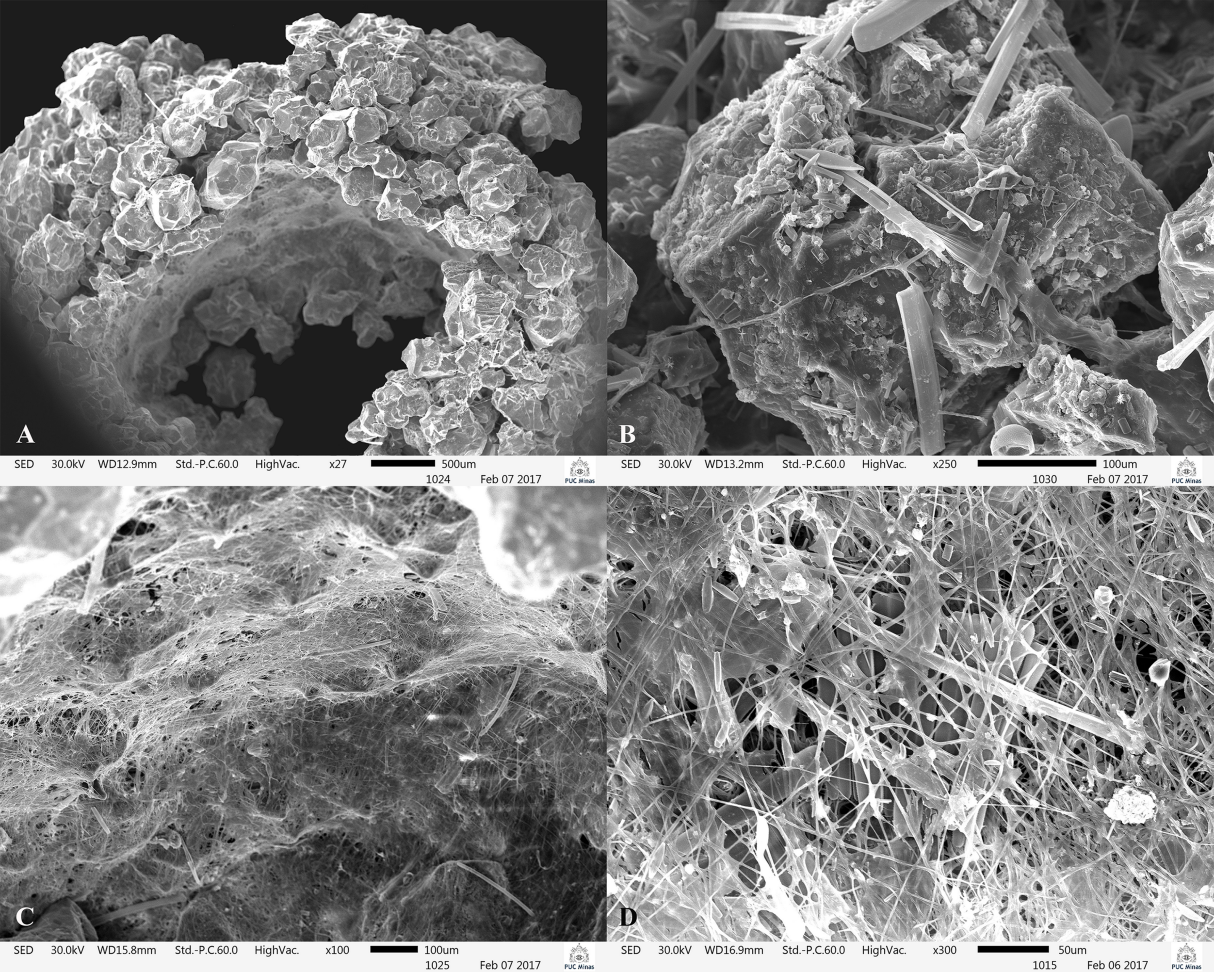
*Plectromacronema solaris* sp. nov. Scanning Electron Microscopy, case and silk. A, case opening. B, case sand grain held by silk. C, silk mat. D, silk mat in detail.

**Diagnosis.** *Plectromacronema solaris* sp. nov. is distinctively different from the 3 previously described species when male genitalic morphology, wing color pattern and pre apical tibial spur formula are analyzed together. *Plectromacronema solaris* sp. nov. male genitalia is similar to *P*. *subfuscum*, however, the well-defined dorsal wart on the Xth segment of *P*. *subfuscum* is not apparent in the new species, which has only some scattered setae in that region. The sclerotized darkened areas of the Xth segment are better defined and uniquely shaped in the new species when compared with the other species in the genus (Figs 5A, 5D and 6A). The globose apex of phallus showing a uniquely shaped concavity with a medial ventral cleft is also diagnostic for *P*. *solaris* sp. nov. (Figs 5B, 5C and 6B). The *P*. *solaris* sp. nov. forewing color pattern resemble *P*. *lisae* apically and *P*. *comptum* medially (Fig 4A and 4B). However, the new species has a forewing color pattern unique for the genus. The new species has a series of clear spots scattered apically and two transversal bars of clear spots medially (Fig 3). The wing venation of the new species also differs from *P*. *comptum* and *P*. *subfuscum*. The crossvein Sc-R is absent in *P*. *solaris* sp.nov. forewings while it is present in *P*. *comptum* and *P*. *subfuscum* (Fig 4C). It should also be noted that the thyridium is present in the new species forewing while this structure is not represented in the illustrations of *P*. *comptum* and *P*. *subfuscum* [10,17]. However the forewing thyridium can be seen on the photography of *P*. *comptum* holotype (Fig 4B). Moreover R4 reaches the discoidal cell in the middle of the crossvein in *P*. *solaris* sp. nov. forewing (Fig 4C) as it also does in *P*. *subfuscum*; in *P*. *comptum* it reaches the base of the crossvein. The hindwing crossvein M2-M3 is present in *P*. *solaris* sp. nov. (Fig 4D) and absent in *P*. *comptum*. The tibial pre-apical spur formula is 2,4,4 which places the new species with *P*. *subfuscum* and *P*. *lisae*, while *P*. *comptum* has 1,4,3 [7] or 2,4,3 [8,10] pre-apical spur formula. The larvae of *Plectromacronema solaris* sp. nov. differs from *P*. *lisae* in the patterns revealed by the brownish markings and muscle scars present on head and notum (Fig 8A). The femur of the foreleg is broader in the new species than in *P*. *lisae* and the trochantin bearing up to five stout setae (Fig 10B), instead of three.

The pupal head of *P*. *solaris* sp. nov. bears mandibles heavily sclerotized, curved inwards, elongated, the vertex of head with about 12 setae as in *P*. *lisae* (Fig 10D). Variation is present in the pupal hook plates in *P*. *solaris* sp. nov. (Fig 11A and 11C), however the shape of the plates on every abdominal segment is different from *P*. *lisae*. Both the shape and number of teeth of the plates differ from *P*. *lisae*. The pupal apical appendages resemble those of *P*. *lisae* but the medial cleft is wider and more squared in the medial section in *P*. *solaris* sp. nov. There is also a unique protuberance facing inwards, bearing setae in the medial section of the apical appendages (Fig 11B).

**Adult.** Forewing length 13.7 mm on the male (n = 1) (Fig 3A and 3C), on female 10.4 mm (n = 7) S1 Table (Fig 3B and 3D). Head dark brown, with patches of white scales dorsally. Compound eyes golden brown. Scape dark brown. Flagellum dark brown extending as much as twice the body length. Maxillary palps golden brown. Prothorax black dorsally, meso- and metathorax golden brown ventrolaterally. Legs golden brown, tibial spur formula 2,4,4. Forewing black, with costal margin ornamented by metallic silver band, entire forewing marked with white spots and metallic golden patches (Fig 3).

**Male genitalia.** Abdominal segment IX anterior margin sinuous when viewed laterally, not produced anteroventrally; posterior margin with dorsolateral keel covered with long setae; as viewed laterally rounded not projecting posteroventrally; as viewed dorsally with short acuminate keel. Segment X covered with small setae dorsolaterally on apex; as viewed dorsally, with bifid round apex. Inferior appendage elongate, with constrictions at 1/4^th^ and 3/4^th^ of its length and with a globose tip, bearing short setae throughout its length (Figs 5A, 5D and 6A). Phallus bent at a right angle basally; apex globose, unarmored. Phallotremal sclerite, as viewed dorsally wing shaped (Figs 5B, 5C and 6B).

**Female genitalia.** Abdominal segment VIII not differentiated, bearing a tuft of setae on posterior margin of sternum; abdominal segment IX anterior margin sinuous when viewed laterally, posterior margin with dorsolateral keel covered with long setae; as viewed laterally rounded not projecting posteroventrally; segment X mostly membranous, setose, with a membranous digitiform projection just above cerci, vaginal apparatus sclerite sinuous as viewed laterally, x shaped as viewed ventrally (Fig 7A and 7B).

**Holotype male** (MUZSP)**. BRAZIL: Minas Gerais:** São Gonçalo do Rio Preto, Parque Estadual do Rio Preto, Córrego das Éguas upstream from Cachoeira dos Crioulos, UV Light trap, 18° 8'47.95"S 43°22'34.93"W, el. 905m, 15.iii.2015, Paprocki et al.

**Paratypes. BRAZIL: Minas Gerais:** São Gonçalo do Rio Preto, Parque Estadual do Rio Preto, Córrego das Éguas upstream from Cachoeira dos Crioulos, UV Light trap, 18°8'47.95"S 43°22'34.93"W, el. 905m, 15.iii.2015, Paprocki et al., 6 females (MUZSP); São Gonçalo do Rio Preto, Parque Estadual do Rio Preto, Rio Preto downstream from Moinho, UV Light trap, 18°5'3.84"S 43°19'49.40"W, el. 749m, 23.iii.2016, Moreira-Silva, Melgaço, Bramuth, 1 female (MZUSP); São Gonçalo do Rio Preto, Parque Estadual do Rio Preto, Rio Preto, Poço dos Veados 18°6'37.16"S 43°20'17.01"W, el. 773m, 24.x.2015, Paprocki & Moreira-Silva, 1 male, 1 female, 1 pharate adult (Larvae collected in the field and reared to adult in laboratory) (MZUSP).

**Larvae.** Length 136mm (n = 27) S1 Table. Larvae is a typical hydropsychid with all notal sclerites completely sclerotized, yellowish with brownish markings and gills present in abdominal segments (Fig 9B). Head longer than wide, yellowish, with brownish and pale markings; clypeus rounded anteriorly with setae, an accessory pair of a small and a large bifurcated setae on anterior third; gena with few small setae, without stridulatory grooves, with a row of short and thick setae along its lateral borders; labrum rounded anteriorly densely covered with setae, even more dense on anterior margin; mandibles well developed with a series of strong, sharp, apical teeth (Fig 10A); submentum convex anteriorly with setae on its lateral border; labium long, elongate and sclerotized.

Thorax with pro, meso and metanotum yellowish with a series of dark and clear markings forming a pattern and bearing a fringe of fine and short hairs, a few long setae scattered (Fig 8A); trochantin broad basally and pointed apically with a fine long setae at the tip, with 5 very thick setae arising dorsally (Fig 10B). Legs nearly equal in length, foreleg femur distinctively wide with rows of long and thick setae on its inner face; tibia and tarsus also with such setae, forming a typical raptorial structure; tarsal claws of forelegs long and slender with single large setae on its base, forming a claw-like structure (Fig 8C). No thoracic gills present. Abdominal gills made of a central stem bearing numerous lateral filaments (Fig 8B), present on abdominal segments I-VIII. Lateral line present on segments 4–8 (Fig 9B). Segments 2–8 with paired ventrolateral pockets densely covered internally with hooked spines (Fig 10C). Oval sclerite on sternum IX bearing long and slender setae on posterior border, covered with thick long setae on surface (Fig 8D). Anal prolegs long and slender, bearing or not a variable papillae dorsally, with long, strong and curved anal claws (Fig 8E).

**Material examined**: São Gonçalo do Rio Preto, Parque Estadual do Rio Preto, Rio Preto, Poço dos Veados, Manual collection, 18°6'37.16"S 43°20'17.60"W, el. 773m, 30.vi.2016, Moreira-Silva & Liberato, 3 larvae; 2.vii.2016, Paprocki & Moreira-Silva, 5 larvae; 17.viii.2016, Paprocki & Moreira-Silva, 2 larvae (MZUSP). São Gonçalo do Rio Preto, Parque Estadual do Rio Preto, Córrego das Éguas, Cachoeira dos Crioulos, Manual collection, 18°8'43.95"S 43°22'5.63"W, el. 896m, 1.vii.2016, Paprocki & Moreira-Silva, 2 larvae (MZUSP), 1 larva (MCN-PUC Minas). São Gonçalo do Rio Preto, Parque Estadual do Rio Preto, Rio Preto, Vau das Éguas, Manual collection, 18°5’55.57”S 43°19’46.98”W, el. 745 m, 30.vi.2016, Moreira-Silva & Liberato, 1 larva (MCN-PUC Minas).

**Distribution.** Brazil (Minas Gerais) (Fig 1).

**Etymology.** The species name refers to the sun bathed pools where the larva is found.

**Biology.** Larvae were dissected and gut contents analyzed (n = 3) under the stereoscopic microscope (magnification: 40X). The gut contents were mostly arthropod parts indicating the predacious habit of the species. Amorphous black matter was also found in gut contents, as mentioned by Flint for *P*. *lisae* [7].

Larvae inhabit tubular retreats built out of silk and sand grains without a capture net (Fig 9A). Length, 36mm, max, 90mm, width 41mm, max 50mm, (n = 267) S1 Table. Larvae were collected in sandy bottom, slow current stretches of streams in depths from 3 to 50 cm (Fig 2). The retreat is lined internally with a mantle of silk tightly woven by the larvae (Fig 12A, 12C and 12D). Uneven grains of sand are glued together with silk to compose the walls of the retreat externally on which diatoms were observed (Fig 12B).

While fed in the laboratory, larvae remained with its posterior end inside of the retreat and attacked the forceps with a rapid forelegs strike, removing the flour and returning to inside of the retreat; a few times the larvae rejected the flour. Larvae spent most of the time inside of the retreat and showed the capacity to turn around inside of it, coming out with either the head or anal prolegs first. Larvae immediately started to rebuild the retreat when displaced. They systematically glued selected grains of sand with silk, building a new retreat in 35–90 minutes. Three pharate adults were encountered floating dead and the emergence of 6 adults happened on 2/11/2015 to 11/02/2016 in the laboratory.

Both in the field or in the water tank in the lab, a network of loose silk threads were placed around the retreat opening, which were colonized by diatoms and possibly other algae and invertebrates. We hypothesized that the structure of the silk nets around the retreat provides a more profitable field for the larvae to encounter its prey than the instable and somehow sterile sandy substrate. Small invertebrates were seen inhabiting these silk nets around retreats in the field. *Plectromacronema solaris* sp. nov. is a suitable model species for ecological and evolutionary studies because its large size, ubiquity of the larvae and biology.

Adults are elusive and were collected only twice out of 12 field trips. Flint [7] also mentioned the majority of adults coming to the light trap were females, which also happened with *P*. *solaris* sp. nov.

## Supporting information

S1 Table Larva length, case length and adults forewing length. (XLSX)
